# Hyperbaric oxygen therapy ameliorates TNBS-induced acute distal colitis in rats

**DOI:** 10.1186/s13618-015-0026-2

**Published:** 2015-04-16

**Authors:** Rogério S Parra, Alexandre H Lopes, Eleonora U Carreira, Marley R Feitosa, Fernando Q Cunha, Sérgio B Garcia, Thiago M Cunha, José J R da Rocha, Omar Féres

**Affiliations:** Division of Coloproctology, Department of Surgery and Anatomy. Ribeirão Preto Medical School, University of São Paulo, Ribeirão Preto, SP Brazil; Department of Pharmacology, Ribeirão Preto Medical School, University of Sao Paulo, Ribeirão Preto, SP Brazil; Department of Pathology, Ribeirão Preto Medical School, University of Sao Paulo, Ribeirão Preto, SP Brazil

**Keywords:** Hyperbaric oxygen, Experimental colitis, Inflammatory bowel diseases, Hypoxia, Cytokines

## Abstract

**Background:**

This study investigated the therapeutic effects of hyperbaric oxygen in experimental acute distal colitis focusing on its effect on the production of pro-inflammatory cytokines, nitric oxide and hypoxia-inducible factor 1alpha.

**Methods:**

Colitis was induced with a rectal infusion of 150 mg/kg of TNBS under anesthesia with Ketamine (50 mg/kg) and Xylazine (10 mg/kg). Control animals received only rectal saline. After colitis induction, animals were subjected to two sessions of hyperbaric oxygen and were then euthanized. The distal intestine was resected for macroscopic analysis, determination of myeloperoxidase activity, western-blotting analyses of inducible nitric oxide synthase and cyclooxygenase-2 expression and immunohistochemical analysis of hypoxia-inducible factor 1alpha and cyclooxygenase-2. Cytokines levels in the distal intestine were measured using an enzyme-linked immunosorbent assay.

**Results:**

Hyperbaric oxygen therapy attenuated the severity of acute distal colitis, with reduced macroscopic damage score. This effect was associated with prevention in the increase of pro-inflammatory cytokine production; myeloperoxidase activity, in the expression of inducible nitric oxide synthase and cyclooxygenase-2. Finally, hyperbaric oxygen inhibited the acute distal colitis-induced up-regulation of hypoxia-inducible factor 1alpha.

**Conclusions:**

The results indicate that hyperbaric oxygen attenuates the severity of acute distal colitis through the down-regulation of pro-inflammatory events.

## Background

The etiology of inflammatory bowel disease (IBD), including Ulcerative colitis (UC) and Crohn’s disease (CD), is still unknown and most likely involves a complex interaction of genetic, environmental, and immune regulatory factors [1-3]. It is proposed that hypoxia and an inappropriate mucosal immune response to normal intestinal constituents are key factors that lead to an imbalance in local pro- and anti-inflammatory cytokines, including a high concentration of tumor necrosis factor-alpha (TNF-α) and interleukin-1 beta (IL-1β) and increased expression of hypoxia-inducible factor 1alpha (HIF-1α) [1,4-6].

The protein encoding HIF-1α has been proposed in the pathogenesis of IBD. It plays an essential role in the cellular and systemic responses to hypoxia. In inflamed mucosa, the oxygen supply is insufficient, which is due to vasculitis and increased oxygen consumption by the inflammatory infiltrate. This low oxygen tension results in HIF-1α stabilization and the activation of the hypoxic adaptive response [7].

In the context of inflammatory diseases, hypoxia has been shown to activate multiple inflammatory mechanisms that are associated with inflammation [8]. Initial observations have indicated that colonic epithelia become severely oxygen deprived during inflammation [9]. Because ulceration and regeneration of the intestinal epithelium occur during the course of the disease, an increased cell metabolism is integral to the pathology of IBD [10]. HIF-1α also controls the aspects of inflammation, including swelling of injured tissues and leukocyte infiltration [11]. The contribution of each of the mediators (pro-inflammatory cytokines, HIF-1α) to the inflammatory cascade remains unknown, but it is conceivable that free radicals and nitric oxide (NO), stimulated by cytokines and HIF-1α, are the final agents responsible for the infliction of tissue damage [7,12].

Evolving therapies for IBD have been promising over the last decade, but some cases still require alternative drugs and supportive therapies. This constant search for new and more effective treatment modalities has generated some promising approaches, such as hyperbaric oxygen (HBO) therapy. A routine HBO therapy consists of the intermittent inhalation of 100% oxygen at pressures greater than those at sea level. Oxygen inhaled at pressures greater than room air pressure dissolves in plasma [13].

There are reports showing that HBO therapy is effective for the treatment of experimental colitis by of trinitrobenzenesulfonic acid–ethanol (TNBS) [12,14,15] and as an adjunctive therapy for healing perianal manifestations of CD [16-18] and UC [19-21]. Despite being beneficial in IBD, the mechanisms responsible for its therapeutic effects have not been elucidated. Thus, the aim of the present study was to investigate the therapeutic effects of HBO on experimentally induced acute distal colitis focusing on its effect on the production of pro-inflammatory cytokines, nitric oxide synthase and HIF-1α.

## Methods

### Animals

This study was approved by the Ethical Committee of Ribeirão Preto Medical School, University of Sao Paulo (n° 54/2009). Twenty-eight *Wistar* rats (male, 150–180 g) were kept in a room with a constant temperature of 22 ± 1°C, with a 12-h/12-h light/dark cycle and were fed standard pellet chow and water ad libitum. The rats were randomly divided into four groups: I- Saline rats, submitted to intracolonic infusion of saline solution (n = 7); II- Saline/HBO rats, submitted to intracolonic infusion of saline solution plus HBO treatment (n = 7); III- TNBS rats, submitted to intracolonic infusion of TNBS (n = 7); IV- TNBS/HBO rats, submitted to intracolonic infusion of TNBS plus HBO treatment (n = 7).

### Induction of colitis

After overnight fasting, distal colitis was induced under light intramuscular anesthesia with 50 mg/kg of Ketamine (Ketalar, Aché Laboratory and Pharmacy, Guarulhos, São Paulo, Brazil) and 10 mg/kg of Xylazine (Dopaser, Calier S.A, Barcelona, Spain) by means of an intrarectal administration of 1 ml of TNBS (Sigma-Aldrich, Deisenhofen, Germany) solution (150 mg/kg) dissolved in 50% ethanol, using an 4 cm-long cannula. Comparisons were carried out with rats that were administered an equal volume (1 ml) of saline solution (Saline and Saline/HBO) as described by other authors [22,23].

### Hyperbaric oxygen

HBO was performed immediately after the induction of colitis and 24 hours after in the Saline/HBO and TNBS/HBO groups. Each session consisted of an exposure of 100% HBO at 2 atmosphere (ATM) for 120 min. Animals in the Saline and TNBS groups remained in the chamber during the time corresponding to a session but were not pressurized.

### Operative technique

**Figure 1:**
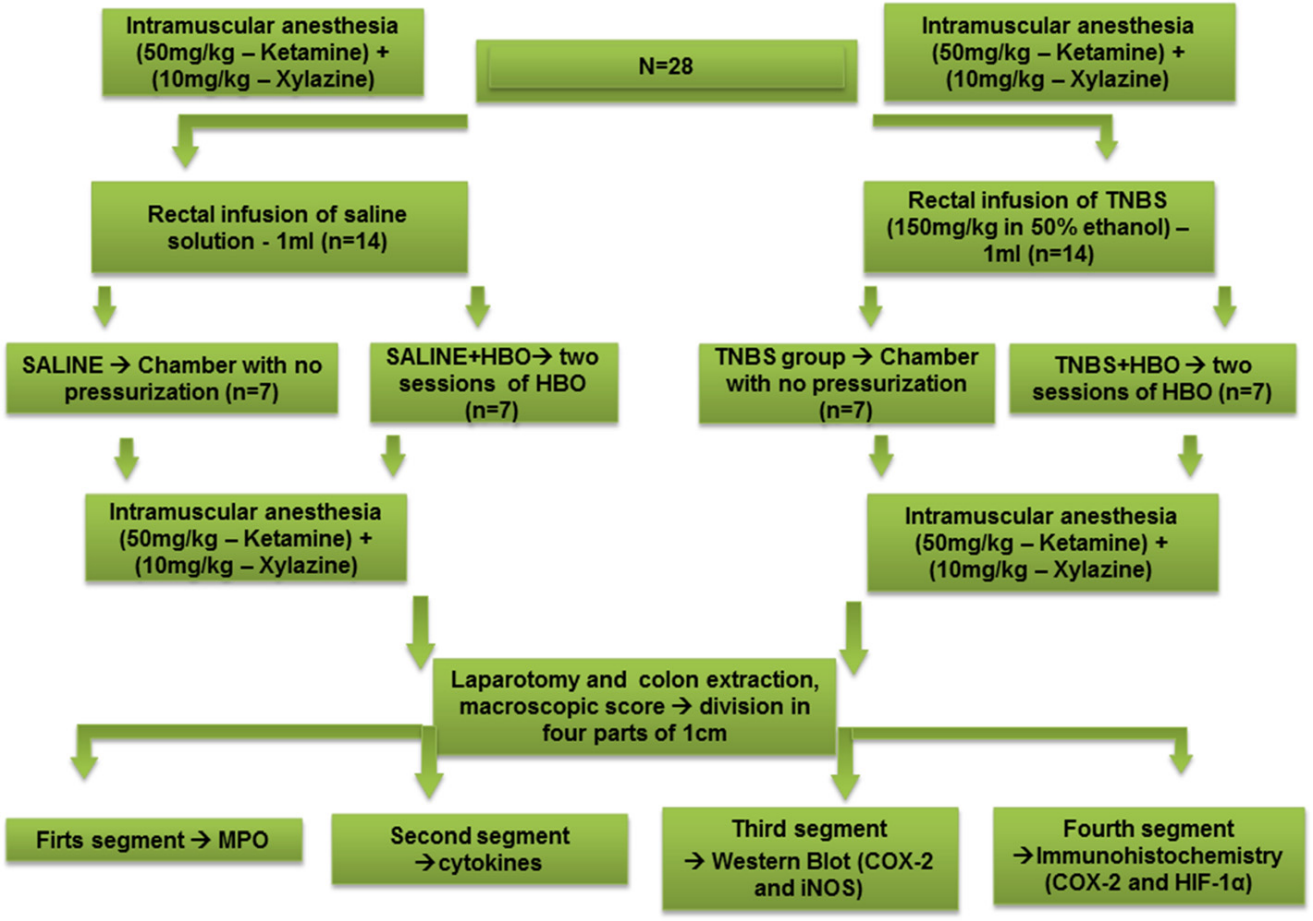
Flow-chart of experiment. All animals (n = 28) were submitted to intramuscular anesthisia (Ketamine 50 mg/kg and Xylazin 10 mg/kg), then divided to four groups: Saline rats (n = 7): submitted to intracolonic infusion of saline solution, no chamber pressurization; Saline/HBO rats: submitted to intracolonic infusion of saline solution plus two sessions of HBO (n = 7); TNBS rats, submitted to intracolonic infusion of TNBS and no chamber pressurization (n = 7); TNBS/HBO rats, submitted to intracolonic infusion of TNBS plus two sessions of HBO (n = 7). After the second HBO session all animals were submitted to intramuscular anesthesia, laparotomy, colon extraction, macroscopic damage score and the colon was divided into four segments, of 1 cm each (First segment: MPO; Second: Cytokines; Third: Western Blot (COX-2 and iNOS); Fourth: Immunohistochemistry (COX-2 and HIF-1α).

After the second HBO session, the rats underwent laparotomy, and the distal colonic segments were taken 6 cm proximal to the anus. The colonic segments were excised longitudinally, rinsed with saline buffer, placed on an ice-cold plate, cleaned of fat and mesentery, and blotted on filter paper. Then, the distal colon was divided in four parts of full-thickness segments of 1 cm on its longitudinal axis. The first segment was used to determine the myeloperoxidase activity (MPO). The second segment was used to evaluate tissue cytokines. The third segment was used for Western blotting. The fourth segment was used for immunohistochemistry. After colon excision, all the animals were euthanized by overdose of Ketamine/Xylazine anesthesia. A flow-chart of the experiment is presented in Figure 1.

### Macroscopic analysis

Macroscopic damage score was performed according the scale previously used for experimental colitis by Gulec et al. [24] (Table 1). A pathologist, without prior knowledge regarding the treatment protocols, examined the 6 cm long distal colon segment immediately after laparotomy to evaluate if there was any focal, multifocal or diffuse ulcer and necrosis. This macroscopic scoring was performed in each rat.

**Table 1:** **The scale of macroscopic damage scoring**

**Macroscopic scoring parameter**	**Score**
*Normal appearance*	0
*Focal ulcer*	1
*Multifocal ulcer*	2
*Diffuse ulcer and necrosis*	3

### Determination of tissue myeloperoxidase activity

Neutrophil accumulation in the colon tissue of rats was evaluated by assaying myeloperoxidase (MPO) activity. Tissue MPO activity was determined as described by Krawisz et al. [25]. Briefly, the tissue samples (250- to 500-mg) were homogenized in 10 vol of cold-potassium buffer (20 mmol/l K2HPO4, pH 7.4). Then, the homogenate was centrifuged at 2000 g for 15 min at 4°C. The pellet was re-homogenized with an equivalent volume of 50 mmol/l K2HPO4 containing 0.5% (w/v) hexadecyltrimethyl-ammonium hydroxide. MPO activity in the suspended pellet was assayed by measuring the change in absorbance at 450 nm using a reading solution (5 mg *O*-dianisidine; 15 μL of 1% H2O2; 3 ml phosphate buVer; 27 ml H2O). The change in absorbance was recorded and plotted on a standard curve of the neutrophil density, with the obtained data expressed as myeloperoxidase activity (neutrophils/mg of tissue).

### Determination of tissue cytokines concentration

The IL-1β, cytokine-induced neutrophil chemoattractant-1 (CINC-1), interleukin-10 (IL-10) and TNF-α levels were quantified in the colon tissues as described previously [26] by using a commercially available enzyme-immunometric assay (ELISA) kit (R&D Systems, Minneapolis USA). The specimen was stored at −70°C until it was required for assay. In brief, the colon specimens were dissected, frozen with liquid nitrogen, crushed in a mortar and pestle, solubilized in phosphate buffered saline (PBS) and measured using ELISA, with the results expressed as picograms per milliliter for each cytokine. As a control, the concentration of each cytokine was determined in the Saline group.

### Western blotting analysis

The specimens were stored at −70°C until required for assay. Colon samples were homogenized in RIPA buffer with a complete protease inhibitor cocktail (Roche). The expression of cyclooxygenase-2 (COX-2; 72 kDa) and inducible nitric oxide synthase (iNOS; 110 kDa) were evaluated by Western blotting analysis. Briefly, the protein concentration was determined following Bradford’s colorimetric method. Proteins were separated by SDS-polyacrylamide gel electrophoresis (SDS-PAGE-12%) and trans blotted onto nitrocellulose membranes (Amersham Pharmacia Biotech, Little Chalfont, UK). After blocking with 5% dry milk (overnight), the membranes were incubated with specific primary antibodies at a dilution of 1:1000 (for COX-2, Abcam, Cambridge, UK and iNOS SIGMA-Aldrich, S. Louis, US). After 3 washes, the filter was then incubated with the secondary horseradish peroxidase-linked anti-goat IgG (for COX-2) and anti-mouse IgG (for iNOS) antibodies. To check for equal loading, the blots were analyzed for β-actin expression. Immunodetection was performed using an enhanced chemiluminescence light-detecting kit (Amersham Pharmacia, Biotech, Little Chalfont, UK). Densitometric data were measured following normalization to the control (house-keeping gene) by Scientific Imaging Systems (Image labTM 3.0 software, Biorad Laboratories, Hercules CA). Data were expressed as the relative density of iNOS/β-Actin bands and COX-2/ β-Actin bands.

### Immunohistochemical analysis

Tissue samples were fixed in 4% neutral formalin and were embedded in paraffin. Immunohistochemical staining was performed using the Biocare Medical Mach 4 Universal Polymer Detection (Concord, CA, USA) kit. The protocol used has been described elsewhere [27]. The dilution and source of the primary antibodies used in this study were HIF-1α (1:50, clone H1alfa67-sup, Abcam, Cambridge, UK) and COX-2 (1:200, clone 4H12, Novocastra®, Newcastle upon Tyne, UK). In accordance with the literature, the immunohistochemistry study was evaluated as follows. For HIF-1α expression, only homogenously and darkly stained nuclei were considered, and cases were considered positive when more than 1% of the colon cells were stained. The slides were evaluated by two experienced pathologists. After immunohistochemistry reactions, the slides were scanned as high-resolution images using Aperio Scan- Scope XT (Aperio, Vista, CA, USA). The images were then visualized using Image Scope software (Aperio, Vista, CA, USA). COX-2 expression in the submucosa was visualized by staining with a rabbit anti-mouse COX-2 polyclonal antibody. The immunoreactivity was considered positive when perinuclear and cytoplasmic cell staining for COX-2 were detected. Quantification of the positively and negatively marked cells was then performed to establish the ratio between these markers.

### Statistical analysis

The results are presented as means ± SEM. The differences were evaluated by one-way ANOVA followed by Bonferroni’s test (three or more groups) or Mann-Whitney’s test (two groups). The level of significance was set at *P* < 0.05.

## Results

### HBO ameliorates acute distal colitis: reduction in the macroscopic damage scores

**Figure 2:**
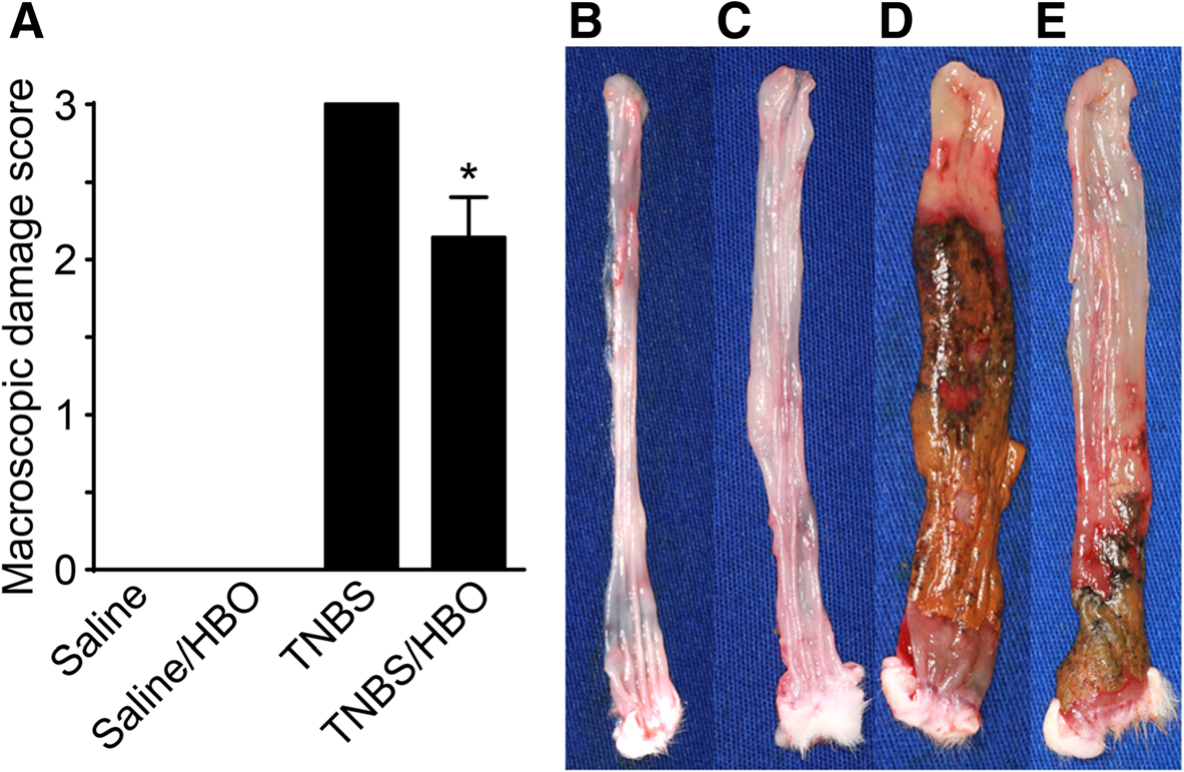
Hyperbaric oxygen therapy ameliorates acute distal colitis by diminishing the macroscopic damage scores. **(A)** Macroscopic damage score. The results are presented as: − Normal Appearance: Score 0; − Focal ulcer: Score 1; − Multifocal ulcer: Score 2; − Diffuse ulcer and necrosis: Score 3. **(B)** Intestine of control rat (Saline), normal appearance (score zero); **(C)** Intestine of Saline/HBO rat, normal appearance (score zero); **(D)** Intestine of TNBS rat, diffuse ulceration and necrosis (Score three); **(E)** Intestine of rat with colitis treated with hyperbaric oxygen (TNBS/HBO) had reduced macroscopic damage score (Score two). Values expressed as mean ± SEM. (n = 7 per group). * P < 0.02 compared to colitis group (Mann–Whitney).

Colitis was confirmed in rats, which received an intrarectal injection of TNBS. These animals presented increased macroscopic damage scores. HBO treatment reduced the colonic damage relative to that of the untreated colitis group (*P* < 0.02). HBO therapy was found to be effective in ameliorating the macroscopic lesion score (Figure 2A). Examples of the distal colon in Saline, Saline/HBO, TNBS and TNBS/HBO are shown in (Figure 2B-E).

### HBO reduces tissue myeloperoxidase activity (MPO) and pro-inflammatory cytokine expression in acute distal colitis

**Figure 3:**
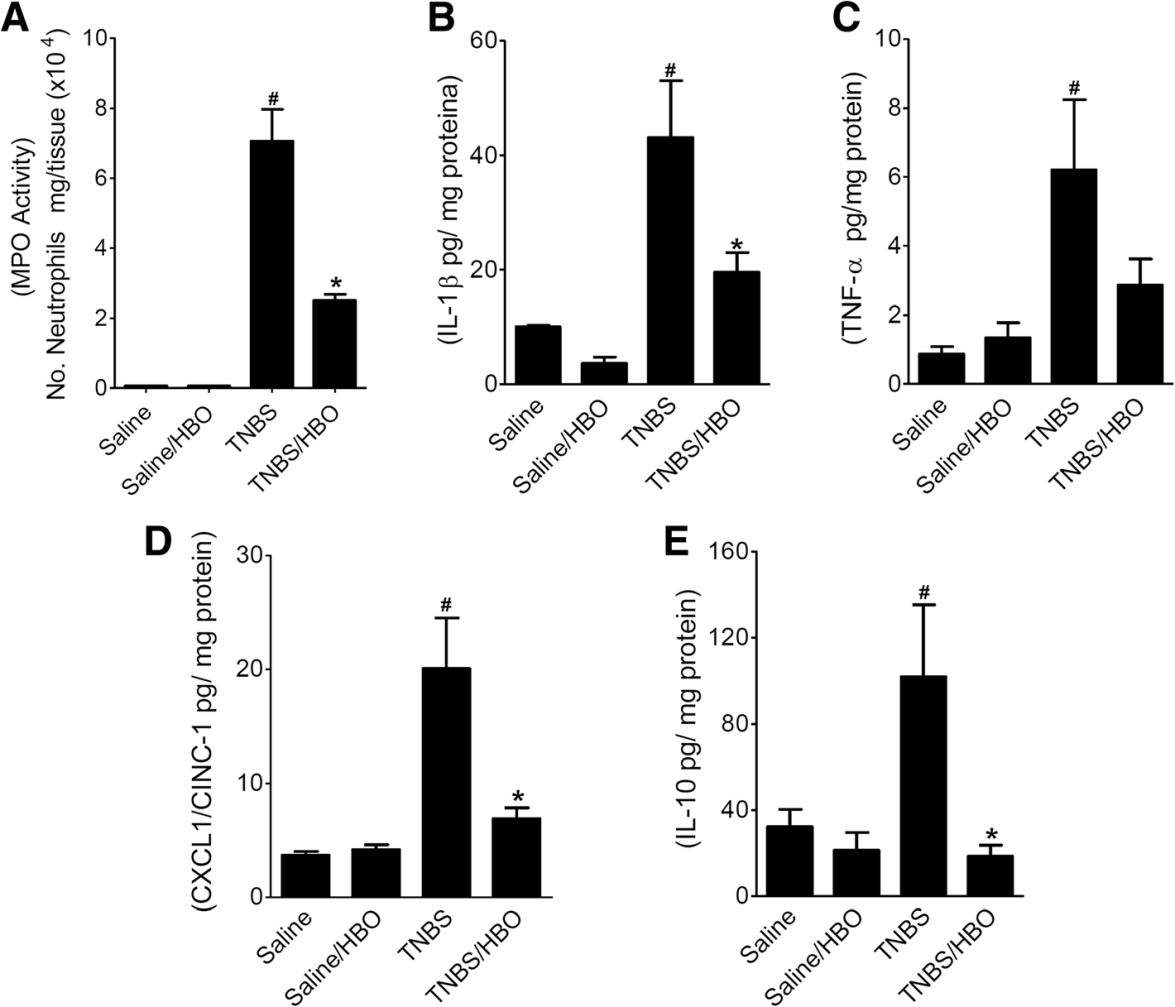
Hyperbaric oxygen reduces tissue myeloperoxidase activity (MPO) and pro-inflammatory cytokine expression in acute distal colitis. **(A)** Acute distal colitis was induced by an intracolonic injection of TNBS (150 mg/kg). After two sessions of hyperbaric oxygen (HBO) the rats were killed, the distal 6 cm colonic segment was removed, and the MPO activity was measured. The anti-inflammatory effect of HBO was detected as a decrease in the number of tissue neutrophils. **(B)** Colitis by TNBS increases the IL-1β tissue expression and HBO decreases its expression. **(C)** Colitis by TNBS increases the TNF-α tissue expression. There was not a significant decrease in expression after HBO. **(D)** Colitis by TNBS increases the CINC-1 tissue expression and HBO decreases its expression. **(E)** Colitis by TNBS increases the IL-10 tissue expression and HBO decreases its expression. Data are expressed as the mean ± S.E.M. (n = 6–7). Data are expressed as the mean ± S.E.M. (n = 6–7). # P < 0.05 compared to the saline-injected group; *P < 0.05 compared to colitis (TNBS) group; (one-way ANOVA followed by the Bonferroni’s test).

Tissue MPO activity was significantly increased in rats with colitis (TNBS) compared to controls (*P* < 0.001), and the HBO treatment (TNBS/HBO) significantly decreased the MPO activity (*P* < 0.001) (Figure 3A). Rats with colitis (TNBS) had significantly increased tissue levels of all cytokines compared to controls (*P* < 0.05). Treatment with HBO normalized IL-1β, TNF-α, CINC-1 and IL-10 (*P* < 0.05) in rats subjected to colitis by TNBS (Figure 3B,C,D and E).

### HBO reduces COX-2, iNOS and HIF-1α expression in acute distal colitis

**Figure 4:**
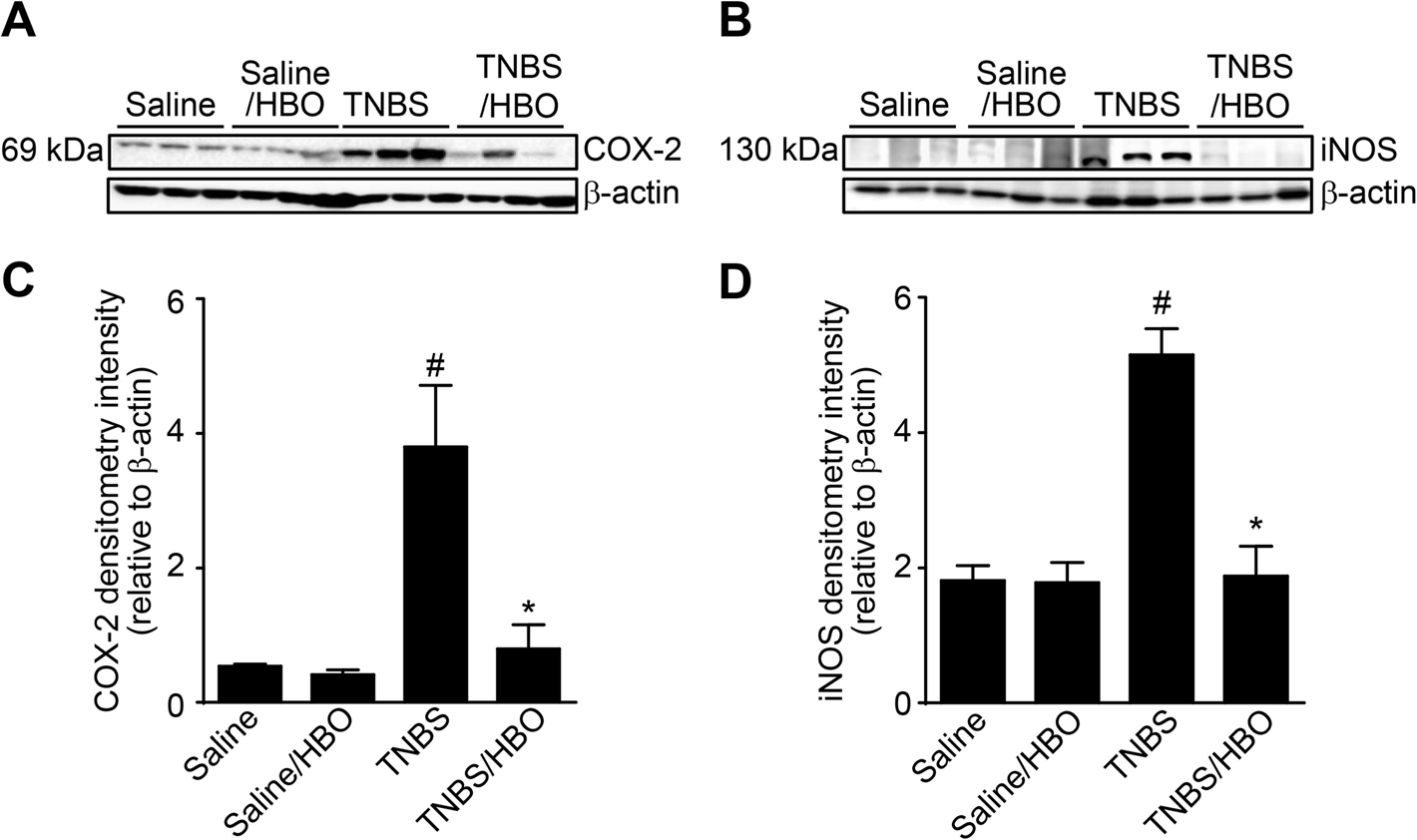
Hyperbaric oxygen reduces tissue COX-2 and iNOS expression. **(A)** Acute distal colitis was induced by the intracolonic injection of TNBS (150 mg/kg). After two sessions of hyperbaric oxygen, the rats were killed, the distal 6 cm colonic segment was removed, and the expression of COX-2 was determined by western blot. The β-Actin level was used as a control. Data are presented as representative blots. **(B)** Densitometry of the pixel intensity of COX-2 bands relative to β-Actin is present. **(C)** Expression of iNOS was determined by western blot. The b-actin level was used as a control. **(D)** Densitometry of the pixel intensity of iNOS bands relative to β-Actin is present. Data are presented as representative blots. Data are expressed as the mean ± S.E.M. (n = 5–7). # *P* < 0.01 compared to the saline-injected group; * *P* < 0.01 compared to colitis (TNBS) group (one-way ANOVA followed by the Bonferroni’s test).

**Figure 5:**
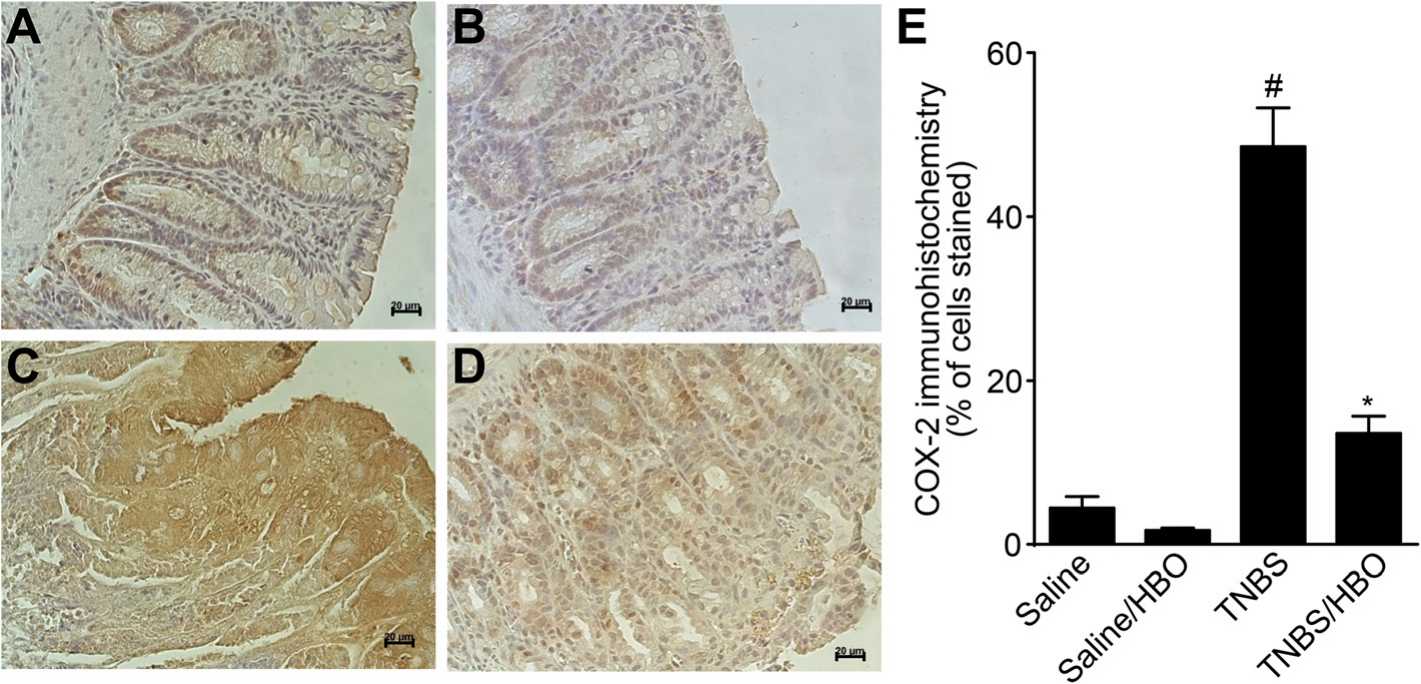
Hyperbaric oxygen reduces COX-2 expression in acute distal colitis. Acute distal colitis was induced by intracolonic injection of TNBS (150 mg/kg). After two sessions of hyperbaric oxygen, the rats were killed, the distal 6 cm colonic segment was removed, and the expression of COX-2 in sub mucosa was determined by immunohistochemical analysis. **(A)** Group I (Saline), **(B)** Group II (Saline/HBO), **(C)** Group III (TNBS), **(D)** Group IV (TNBS/HBO), **(E)** Rats with colitis (TNBS) presented substantial up-regulation of immunofluorescence of COX-2 in the colon. The HBO treatment (TNBS/HBO) remarkably reduces COX-2 expression. Data are expressed as the mean ± S.E.M. (n = 7). # *P* < 0.001 compared to the saline-injected group; *P < 0.001 compared to colitis (TNBS) group (one-way ANOVA followed by the Bonferroni’s test).

**Figure 6:**
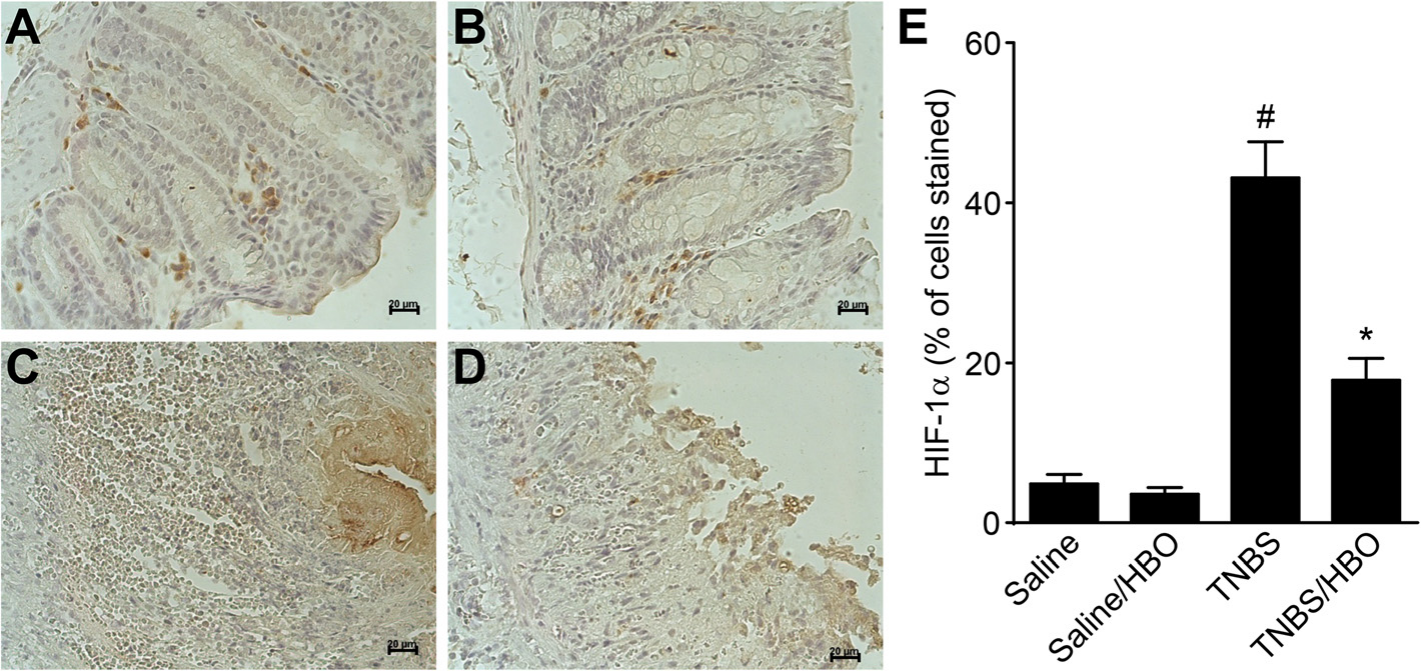
Hyperbaric oxygen reduces HIF-1α expression in acute distal colitis. Acute distal colitis was induced by intracolonic injection of TNBS (150 mg/kg). After two sessions of hyperbaric oxygen, the rats were killed, the distal 6 cm colonic segment was removed, and the expression of HIF-1α was determined by immunohistochemical analysis. **(A)** Group I (Saline), **(B)** Group II (Saline/HBO), **(C)** Group III (TNBS), **(D)** Group IV (TNBS/HBO), and **(E)** Rats with colitis (TNBS) presented substantial up-regulation of immunofluorescence of HIF-1α in the colon. The HBO treatment (TNBS/HBO) remarkably reduces HIF-1α expression. Data are expressed as the mean ± S.E.M. (n = 7). # P < 0.001 compared to the saline-injected group; *P < 0.001 compared to colitis (TNBS) group (one-way ANOVA followed by the Bonferroni’s test).

Western blotting analysis of intestinal extracts showed that colitis rats (TNBS) had significantly increased COX-2 and iNOS protein expression compared to controls (*P* <0.01), and there was a significant decrease in the relative density of COX-2 and iNOS protein in colitis rats treated with HBO (TNBS/HBO) (*P* < 0.01) (Figure 4A and C). The relative analysis of the COX-2, iNOS and β-actin expression are shown in Figure 4B and D. Immunohistochemical analysis of the intestinal extract showed that TNBS rats had increased COX-2 and HIF-1α tissue expression compared to controls (*P* < 0.001), and there was a significant decrease in COX-2 (Figure 5A-E) and HIF-1α (Figure 6A-E). in rats with colitis treated with HBO (TNBS/HBO) (*P* < 0.001).

## Discussion

Despite the growing number of therapeutic methods and the recent application of new drugs for the treatment of IBD, many patients still present with refractory symptoms. The HBO is an interesting therapeutic approach although its mechanism of action is not totally clear. The present results demonstrate effectiveness of HBO therapy in a model of experimental acute distal colitis in rats by decreasing tissue damage. In addition, the expression levels of major inflammatory mediators in the damage tissue such as pro-inflammatory cytokines, HIF-1α, iNOS and COX-2 as well as neutrophil infiltration were found to be down-modulated by hyperbaric oxygenation, suggesting that they were involved in the therapeutic effects in experimental acute colitis.

In the present study, neutrophils infiltration into intestinal lesions was indirectly evaluated by the myeloperoxidase activity assay. MPO, an enzyme found primarily within neutrophils, is a sensitive marker for quantifying neutrophil content in tissues. The beneficial effects of HBO may be partially attributed to its ability to reduce neutrophil activation and sequestration in inflammatory intestinal mucosa.

A large number of evidences have revealed that the increase of oxidative stress and iNOS activity was a notable feature of IBD, which resulted in a pathological cascade of free radical reactions and further yielding more oxidative free radicals to impair the structure and function of cell [28,29]. As we know small amounts of NO are necessary to maintain tissue integrity. However, hypoxia, HIF-1α and pro-inflammatory cytokines (mainly IL-1β and TNF-α) activation leads to increased iNOS expression and excessive NO production by macrophages, resulting in tissue injury. Such damage further enhances cytokine release by inflammatory cells in a feedback loop, restarting the cycle [26].

In this present study, COX-2 levels were higher in TNBS-induced rats and HBO was able to reduce their levels. It is known that abnormal metabolism of arachidonic acid is another vital factor in the IBD pathogenesis. COX-2 could be activated to produce excessive prostaglandin E2 and thromboxane B2, two important inflammatory mediators, in the inflammatory bowel disease, which contribute to bowel hyperemia, edema and even dysfunction. In addition, thromboxane B2 could also induce platelet aggregation, vasoconstriction and microthrombosis, aggravating the inflammatory reaction [30].

Accordingly, a possible mechanism to explain the effect of HBO therapy after TNBS-induced in rats is increase tissue oxygen (consequently decreases HIF-1α) and reduced production of IL-1β, CINC-1 and TNF-α in the local tissue. This data are consistent with new studies that showed the correlation between an inflammatory signals induce IL-1β through HIF-1α [31]. Therefore, one study suggests that the reduction of IL-1β production may play an important role in the immunosuppressive effect of HBO, as shown by other authors [32].

A number of studies suggest a close association between inflammation and hypoxia at the tissue level, resulting in increased levels of HIF-1α [3,33-39]. Herein, it was provided evidence that HBO therapy is essential to control the level of HIF-1α and therefore improve colitis by TNBS-induced. Another studies from both human disease [40] and experimental colitis [34,36] suggest the presence of colonic hypoxia and its significance in the disease process and indicate that colonic epithelia becomes severely oxygen deprived during inflammation and that epithelial hypoxia in TNBS colitis is associated with inflammatory lesions. Hypoxia cause ischemia which could worsen the disease and to promote necrosis, inflammation, and ulceration in the gut. The HBO therapy diminishes colitis activity by increasing tissue O2 diffusion.

It was proposed that the increased levels of HIF-1α and cytokines (such as IL-1β, TNF-α and CINC-1) contributed significantly to the establishment and maintenance of chronic inflammation [31,35]. We hypothesized that TNBS colitis would result in HIF-1α activation, particularly within the epithelium, resulting in increased expression of pro-inflammatory cytokines such as IL-1β and TNF-α, and higher levels of iNOS. We observe that the expression of iNOS shows significant increase that supposedly trigger by IL-1β and TNF-α. Some works focus in demonstrate the correlation between pro inflammatory cytokines inducing the expression of iNOS protein [41,42]. Increased iNOS activity and, consequently, higher levels of NO, contribute to inflammation, oxidative stress and tissue damage.

Although the present results strongly suggest that HBO might be an interesting alternative therapy for TNBS-induced distal colitis, there are limitations to this study. The exactly mechanism of action of HBO was not elucidated, spite of the results indicate evolvement of pro-inflammatory cytokines and HIF-1α in colonic damage. Measurements were performed only one time (24 hours after colitis induction). And further work is needed to elucidate why TNF-α concentration did not significantly decreased in animals submitted to HBO. Probably at the time this cytokine was measured the higher amount of TNF-α was lost. It is well known that the higher concentration is in the initial phase and after this cytokine is rapidly degraded.

According to the medical literature and our results, we may state the way in which hyperbaric oxygen ameliorates TNBS-induced acute distal colitis as follows: TNBS induces colitis by breaking the mucosal integrity and subsequent damage to intestinal epithelium. There is epithelial hypoxia and induction of HIF-1α, with increased expression of pro-inflammatory cytokines such as IL-1β and TNF-α, and higher levels of iNOS. Higher cytokine and NO levels contribute to inflammation and oxidative stress, neutrophil infiltration which result in mucosal damage, restarting the cycle. Hyperbaric oxygen acts diminishing hypoxia (and HIF-1α), consequently decreasing cytokine expression, NO production, COX-2 and neutrophil infiltration, resulting in less damage e amelioration of colitis.

## Conclusion

The present results strongly suggest that the treatment with HBO ameliorates TNBS-induced model of colitis and is associated with decreased in the severity of inflammation as measured by MPO, cytokine levels, iNOS and COX-2 expression. Furthermore, it is plausible to suggest that the mechanism of the HBO therapy might be through down-regulation of HIF-1α expression. Further studies need to be done to prove this hypothesis.

## Abbreviations

IBD: Inflammatory bowel disease
UC: Ulcerative colitis
CD: Crohn’s disease
TFN-α: Tumor necrosis factor-alpha
IL-1β: Interleukin-1 beta
HIF-1α: Hypoxia-inducible factor 1alpha
NO: Nitric oxide
HBO: Hyperbaric oxygen
TNBS: Trinitrobenzenesulfonic acid–ethanol
ATM: Atmosphere
MPO: Myeloperoxidase activity
CINC-1: Cytokine-induced neutrophil chemoattractant
IL-10: Interleukin-10
ELISA: Enzyme-immunometric assay
PBS: Phosphate buffered saline
COX-2: Cyclooxygenase-2
iNOS: Inducible nitric oxide synthase

## References

[1] Podolsky DK (2002). Inflammatory bowel disease. N Engl J Med.

[2] Shih DQ, Targan SR, McGovern D (2008). Recent advances in IBD pathogenesis: genetics and immunobiology. Curr Gastroenterol Rep.

[3] Taylor CT, Colgan SP (2007). Hypoxia and gastrointestinal disease. J Mol Med.

[4] Higashiyama M, Hokari R, Hozumi H, Kurihara C, Ueda T, Watanabe C (2012). HIF-1 in T cells ameliorated dextran sodium sulfate-induced murine colitis. J Leukoc Biol.

[5] Shah YM, Ito S, Morimura K, Chen C, Yim SH, Haase VH (2008). Hypoxia-inducible factor augments experimental colitis through an MIF-dependent inflammatory signaling cascade. Gastroenterol.

[6] Nandi J, Saud B, Zinkievich JM, Yang ZJ, Levine RA (2010). TNF-alpha modulates iNOS expression in an experimental rat model of indomethacin-induced jejunoileitis. Mol Cell Biochem.

[7] Devisscher L, Hindryckx P, Olivier K, Peeters H, Vos M, Laukens D (2011). Inverse correlation between metallothioneins and hypoxia-inducible factor 1 alpha in colonocytes and experimental colitis. Bioch Biophys Res Commun.

[8] Clambey ET, Mcnamee EM, Westrich JA, Glover LE, Campbell EL, Jedlicka D (2012). Hypoxia-inducible factor-1 alpha-dependent induction of FoxP3 drives regulatory T-cell abundance and function during inflammatory hypoxia of the mucosa. Proc Natl Acad Sci U S A.

[9] Karhausen J, Furuta GT, Tomaszewski JE, Johnson RS, Colgan SP, Haase VH (2004). Epitelial hypoxia-inducible factor-1 is protective in murine experimental colitis. J Clin Invest.

[10] Giatromanolaki A, Sivridis E, Maltezos E, Papazoglou D, Simopoulos C, Gatter KC (2003). Hypoxia inducible factor 1alpha and 2alpha overexpression in inflammatory bowel disease. J Clin Pathol.

[11] Bai X, Sun B, Pan S, Jiang H, Wang F (2009). Down-regulation of hypoxia-inducible factor alpha by hyperbaric oxygen attenuates the severity of acute pancreatitis in rats. Pancreas.

[12] Rachmilewitz D, Karmeli F, Okon E, Rubenstein I, Better OS (1998). Hyperbaric oxygen: a novel modality to ameliorate experimental colitis. Gut.

[13] Feldmeier JJ, Hampson NB (2002). A systematic review of the literature reporting the application of hyperbaric oxygen prevention and treatment of delayed radiation injuries: an evidence based approach. Undersea Hyperb Med.

[14] Akin ML, Gulluoglu BM, Uluutku H, Erenoglu C, Elbuken E, Yildirim S (2002). Hyperbaric oxygen improves healing in experimental rat colitis. Undersea Hyperb Med.

[15] Atug O, Hamzaoglu H, Tahan V, Alican I, Kurtkaya O, Elbuken E (2008). Hyperbaric oxygen therapy is as effective as dexametasone in the treatment of TNBS-E-induced experimental colitis. Dig Sci.

[16] Colombel JF, Mathieu D, Bouault JM, Lesage X, Zavadil P, Quandalle P (1995). Hyperbaric oxygenation in severe perineal Crohn’s disease. Dis Colon Rectum.

[17] Iezzi LE, Feitosa MR, Medeiros BA, Aquino JC, Almeida AL, Parra RS (2011). Crohn’s disease and hyperbaric oxygen therapy. Acta Cir Bras.

[18] Lavy A, Weisz G, Adir Y, Ramon Y, Melamed Y, Eidelman S (1994). Hyperbaric oxygen for perianal Crohn’s disease. J Clin Gastroenterol.

[19] Buchman AL, Fife C, Torres C, Smith L, Aristizibal J (2001). Hyperbaric oxygen therapy for severe ulcerative colitis. J Clin Gastroenterol.

[20] Gürbüz AK, Elbüken E, Yazgan Y, Yildiz S (2003). A different therapeutic approach in patients with severe ulcerative colitis: hyperbaric oxygen treatment. South Med J.

[21] Assche GV, Vermeire S, Rurgeerts P (2011). Management of acute severe ulcerative colitis. Gut.

[22] Elson CO, Cong Y, McCracken VJ, Dimmitt RA, Lorenz RG, Weaver CT (2005). Experimental models of inflammatory bowel disease reveal innate, adaptive, and regulatory mechanisms of host dialogue with microbiota. Immunol Rev.

[23] Neurath MF, Fuss I, Kelsall BL, Stüber E, Strober W (1995). Antibodies to interleukin 12 abrogate established experimental colitis in mice. J Exp Med.

[24] Gulec B, Yasar M, Yildiz S, Oter S, Akay C, Deveci S (2004). Effect of hyperbaric oxygen on experimental acute distal colitis. Physiol Res.

[25] Krawisz JE, Sharon P, Stenson WF (1984). Quantitative assay for acute intestinal inflammation based on myeloperoxidase activity: assessment of inflammation in rat and hamster models. Gastroenterol.

[26] Cunha TM, Verri WA, Silva JS, Poole S, Cunha FQ, Ferreira SH (2005). A cascade of cytokines mediates mechanical inflammatory hypernociception in mice. Proc Natl Acad Sci.

[27] Ribeiro-Silva A, Moutinho MA, Moura HB, Vale FR, Zucoloto S (2006). Expression of checkpoint kinase 2 in breast carcinomas: correlation with key regulators of tumor cell proliferation, angiogenesis, and survival. Histol Histopathol.

[28] Fukumura D, Kashiwagi S, Jain RK (2006). The role of nitric oxide in tumor progression. Nat Rev Cancer.

[29] Pavlick KP, Laroux FS, Fuseler J, Wolf RE, Gray L, Hoffman J (2002). Role of reactive metabolities of oxygen and nitrogen in inflammatory bowel disease. Free Radic Biol Med.

[30] Karmeli F, Cohen P, Rachmilewitz D (2002). Cyclo-oxygenase-2 inhibitors ameliorate the severity of experimental colitis in rats. Eur J Gastroenterol Hepatol.

[31] Tannahill GM, Curtis AM, Adamik J, Palsson-McDermott EM, McGettrick AF, Goel G (2013). Succinate is an inflammatory signal that induces IL-1β through HIF-1α. Nature.

[32] Weisz G, Lavy A, Adir Y, Melamed Y, Rubin D, Eidelman S (1997). Modification of in vivo and in vitro TNF-alpha, IL-1 and IL-6 secretion by circulating monocytes during hyperbaric oxygen treatment in patients with perianal Crohn’s disease. J Clin Immunol.

[33] Semenza GL (2000). HIF-1 and human disease: one highly involved factor. Genes Dev.

[34] Kruschewski M, Foitzik T, Perez-Canto A, Hubotter A, Buhr HJ (2001). Changes of colonic mucosal microcirculation and histology in two colitis models. Dig Dis Sci.

[35] Karhausen J, Haase VH, Colgan SP (2005). Inflammatory hypoxia role of hypoxia-inducible factor. Cell Cycle.

[36] Hauser CJ, Locke RR, Kao HW, Patterson J, Zipser RD (1988). Visceral surface oxygen tension in experimental colitis in the rabbit. J Lab Clin Med.

[37] Suski MD, Zabel D, Levin V, Scheuenstuhl H, Hunt TK, Nemoto EM, LaManna JC (1997). Effect of hypoxic hypoxia on transmural gut and subcutaneous tissue oxygen tension. Oxygen transport to tissue XVIII.

[38] Ahlqvist J (1984). A hypothesis on the pathogenesis of rheumatoid and other non-specific synovitides. IV A. The possible intermediate role of local hypoxia and metabolic alterations. Med Hypotheses.

[39] Eltzschig HK, Carmeliet P (2011). Hypoxia and inflammation. N Engl J Med.

[40] Wakefield AJ, Sawyerr AM, Dhillon AP, Pittilo RM, Rowles PM (1989). Pathogenesis of Crohn’s disease: multifocal gastrointestinal infarction. Lancet.

[41] Kankuri E, Hämäläinen M, Hukkanen M, Salmenperä P, Kivilaakso E, Vapaatalo H (2003). Supression of pro-inflammatory cytokines release by selective inhibition of indutible nitric oxide synthase in mucosal explants from patients with ulcerative colitis. Scand J Gastroenterol.

[42] Ercin CN, Yesilova Z, Korkmaz A, Ozcan A, Oktenil C, Uygun A (2009). The effect of iNOS inhibitors and hyperbaric oxygen treatment in a rat model of experimental colitis. Dig Dis Sci.

